# GNRH1 and LTB4R might be novel immune-related prognostic biomarkers in clear cell renal cell carcinoma (ccRCC)

**DOI:** 10.1186/s12935-021-02052-1

**Published:** 2021-07-06

**Authors:** Hua-Hui Wu, Xin Yan, Zhao Chen, Guo-Wei Du, Xiao-Jie Bai, Kurerban Tuoheti, Tong-Zu Liu

**Affiliations:** Department of Urology, Zhongnan Hospital of Wuhan University, 169 Donghu Road, Wuhan, 430071 China

**Keywords:** Immune-related genes, Clear cell renal cell carcinoma, Prognostic biomarkers, Immune infiltration, WGCNA

## Abstract

**Background:**

Clear cell renal cell carcinoma (ccRCC) occupied most of renal cell carcinoma (RCC), which associated with poor prognosis. The purpose of this study is to screen novel and prognostic biomarkers for patients with ccRCC.

**Methods and results:**

Firstly, Gene Expression Omnibus database was used to collect microarray data for weighted gene co-expression network construction. Gene modules related to prognosis which interest us most were picked out. 90 hub genes were further chosen in the key modules, two of which including gonadotropin releasing hormone 1 (GNRH1) and leukotriene B4 receptor (LTB4R) were screened and validated as immune-related prognostic biomarkers. Based on several public databases and ccRCC tissues collected by ourselves, we performed survival analysis, spearman correlation analysis, receiver operating characteristic (ROC) analysis, quantitative real-time PCR (qRT-PCR), western blotting, immunofluorescence (IF) and immunohistochemistry (IHC) staining for the validation of immune-related prognostic biomarkers. We further explored the relationship between immune-related prognostic biomarker expressions and immunocytes. Finally, gene set enrichment analysis (GSEA) demonstrated that the two immune-related prognostic biomarkers were significantly correlated with cell cycle.

**Conclusions:**

Generally speaking, the present study has identified two novel prognostic biomarkers for patients with ccRCC, which showed strong correlation with prognosis of patients with ccRCC, could further be used as potential prognostic biomarkers in ccRCC.

**Supplementary Information:**

The online version contains supplementary material available at 10.1186/s12935-021-02052-1.

## Background

RCC is an adenocarcinoma derived from renal tubular epithelial cells, 85% of which are ccRCC [1]. ccRCC occupied poor prognosis [1, 2]. There will be approximately 152,160 RCC cases diagnosed and 27,560 RCC-related deaths in the United States in 2021 [2]. The prognosis of ccRCC is influenced by mechanisms covering complex networks of gene interactions [3]. What is worse, ccRCC is insensitivity to both radiotherapy and chemotherapy [3, 4]. Therefore, because of the poor prognosis and the lack of new therapeutic target, we aim to screen out novel and specific prognostic markers for patients with ccRCCs in the present study.

Immunotherapy is a type of cancer treatment, which helps immune system fight cancer [5]. Nowadays, more and more studies have indicated that tumors could be treated by immunotherapy effectively and safely [6, 7]. Thus, identification of immune-related prognostic biomarkers has been one of the research hotspots in cancer treatment.

Thus, in the present study, based on gene expression profiles collected from The Cancer Genome Atlas (TCGA) database, Gene Expression Omnibus (GEO) database, and AffyExpress database (including large size of gene expression matrix about cancers, which have been widely used in gene exploring), we attempted to screen out several immune-related prognostic biomarkers which might occur the potential of prognosis prediction and treatment. To the best of our knowledge, this study might be one of the first studies to identify immune-related prognostic biomarkers in ccRCC directly. Some widely used methods such as weighted gene co-expression network analysis (WGCNA) were also applied in this study to identify two immune-related modules and further screen out two novel immune-related prognostic biomarkers. What we should mention was that we creatively defined prognosis-related modules as modules associated with not only survival time but also survival status. Previous studies also defined prognosis-related modules as modules only associated with one of the two traits (survival time or survival status). But we realized that both of survival status and survival time were important indispensable yardsticks of prognosis. Therefore, two modules associated with both the two traits were preliminarily selected in the present study by WGCNA. Two immune-related genes (IRGs) in the prognosis-related modules were further identified and validated as potential prognostic biomarkers of patients with ccRCC.

To sum up, the present study indicated that two IRGs might be novel immune-related prognostic biomarkers for ccRCC. The two IRGs were validated to show strong potential for prognosis prediction and treatment of ccRCC.

## Materials and methods

### Datasets and immune-related genes (IRGs)

**Fig. 1:**
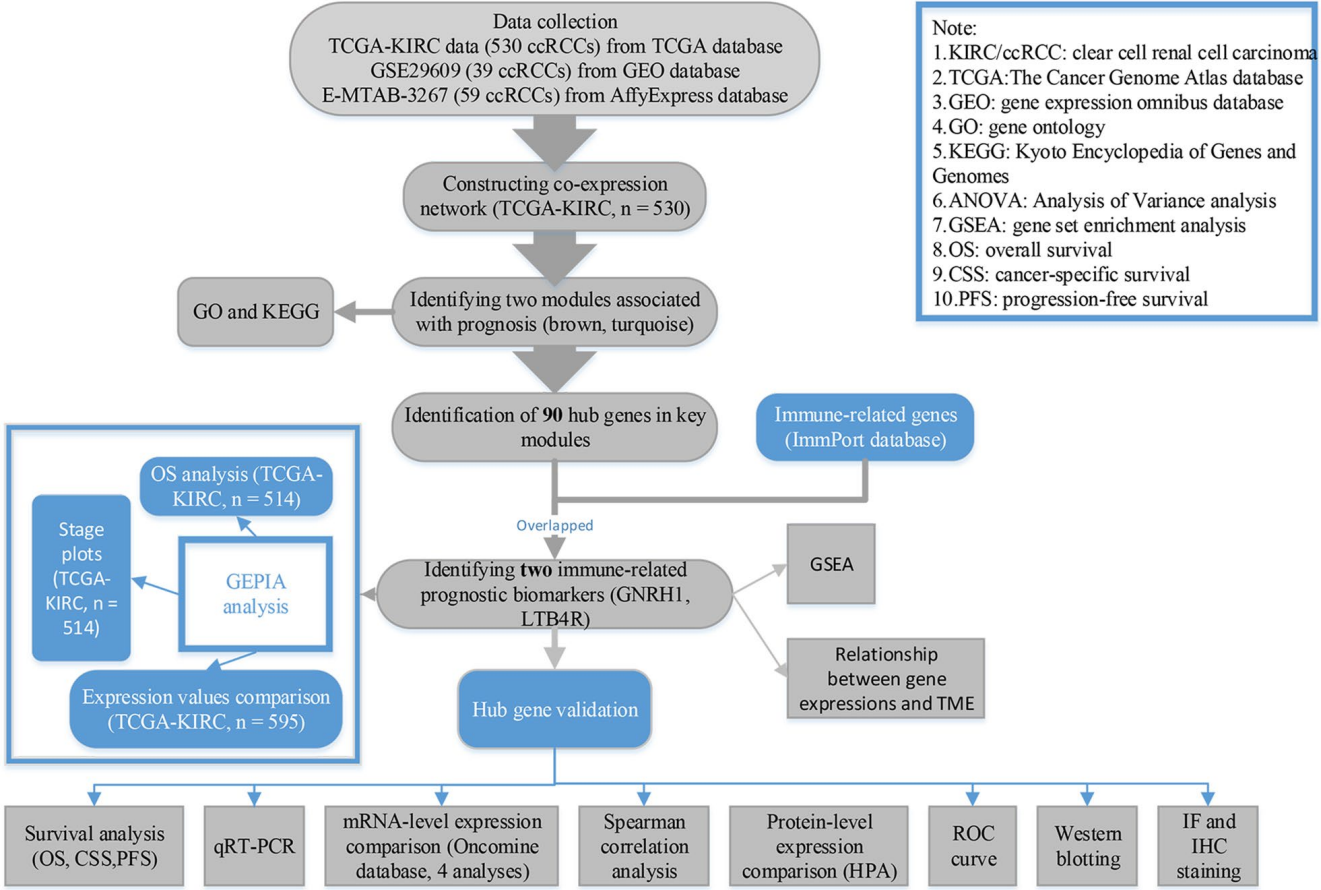
Flow diagram of data preparation, processing, analysis, and validation in this study

Figure 1 showed the workflow of the present study, describing the whole process of IRGs correlated to prognosis exploring. Raw expression data of ccRCC (TCGA-KIRC) were retrieved from TCGA database (https://genomecancer.ucsc.edu/). This expression data was firstly showed as count number. Standardization of TCGA-KIRC data were conducted using R package “DEseq.2” [8]. Generally, we also conducted normalization and log2 transformation via this tool. Finally, 530 ccRCCs containing necessary clinical feature information were chosen, which were further used for WGCNA. Furthermore, GSE29609 [9] including 39 tumor samples was retrieved from Gene Expression Omnibus (GEO) database [10] (http://www.ncbi.nlm.nih.gov/geo/), which was used as a validation cohort. Complete survival information for this dataset was also downloaded in our study. This dataset was normalized and log2 transformed based on R package “affy” [11]. In addition, we directly collected 53 ccRCCs which stored in E-MTAB-3267 from AffyExpress database (https://www.ebi.ac.uk/arrayexpress/) to further verify our results.

Moreover, immune-related genes (IRGs) were collected from the ImmPort database (https://www.immport.org). In total, 2499 IRGs were retrieved, 1811 of which overlapped with gene list of TCGA-KIRC expression matrix were further included in the present study.

### Construction of co-expression network

Firstly, we checked the expression matrix of all genes based on gsg (goodSamplesGenes) and sample network methods in R package “WGCNA” [12]. A standard of Z.Ku ≥ − 2.5 was set to pick out qualified ccRCC samples for constructing a co-expression network. A method called scale free topology criterion was used to pick out approriate β (soft threshold power beta) in WGCNA. In addition, we transformed adjacency into TOM and further classified genes into gene modules. Manual (interactive) branch cutting approach, automatic single block analysis and 2 block analysis were applied for gene module identification [13]. In the three methods, we set the same cut-off criteria as follows: a relatively large minimum module size of minClusterSize = 30, and a medium sensitivity (deepSplit = 2). To merge modules with high correlation, a cut line was also set by reckoning dissimilarity of module eigengenes (MEs).

### Prognosis-related module identification

In the present study, both of survival status and survival time are regarded as important yardsticks of prognosis. Thus, we attempted to screen out hub modules, which associated with both the traits. Gene Significance (GS) which could quantify the association between genes and trait was firstly explored. Module significance (MS) was secondly defined based on GS. By doing these, modules which met the following criteria were regarded as prognosis-related modules: (1) module must show significant association with both survival status [overall survival status (OS)] and survival time (OS time); (2) module showed the highest MS of OS or OS time. We then obtained the Gene Ontology (GO) enrichment analysis and Kyoto Encyclopedia of Genes and Genomes (KEGG) pathway analysis for genes in prognosis-related modules based on R package “clusterProfiler” [14]. In the present study, we thought biological processes (BPs) and KEGG pathway terms with P < 0.05 were functional enriched.

### Novel immune-related prognostic biomarker identification

We immediately screened out hub genes in WGCNA by using the standards of (|cor.geneModuleMembership| > 0.8 and |cor.geneTraitSignificance| > 0.2). Finally, genes overlapped between hub genes in WGCNA and IRGs were considered as immune-related prognostic biomarker, which were validated in subsequent analysis.

### Internal validation of immune-related prognostic biomarkers

Two survival types including overall survival (OS) and disease-free survival (DFS) were conducted by using GEPIA webtool [15], as a preliminary exploration of the prognostic value of immune-related prognostic biomarkers (http://gepia.cancer-pku.cn/). Furthermore, immune-related prognostic biomarker expression levels between normal samples and PAAD samples were compared as an internal validation. Statistical significance was measured by using unpaired *t* test. Besides, stage plots were plotted to measure the difference of hub gene expression in different stages. The statistical significance of stage plots was measured by using ANOVA.

### Prognostic value of immune-related prognostic biomarkers validation

Furthermore, to better understand the prognostic value of novel biomarkers, we used 3 analysis methods in this step. Firstly, by using GSE29609 (n = 39) and E-MTAB-3267 (n = 53), ccRCCs in the two datasets were grouped into high- and low- expression groups via the most appropriate score cutoff for tissues splitting calculated using R package “maxstat” [16]. We immediately conducted three kinds of survival analyses (OS, CSS, PFS) via R package “survival” [17]. Secondly, by using TCGA-KIRC data, receiver operating characteristic (ROC) curves were drawn to see if immune-related prognostic biomarkers could distinguish ccRCC tissues from normal tissues. ROC analysis was conducted based on R package “pROC” [18]. Furthermore, to measure the resolving power, we calculated the area under curve (AUC). In this study, we considered an immune-related prognostic biomarker with AUC > 0.75 had strong prognostic value. Thirdly, spearman correlation analysis was obtained based on SPSS (version 21.0), R package “ggstatsplot” [19] was used for visualization.

### mRNA expression level and translation-level expression of prognostic biomarker verification

After an internal validation by using GEPIA, we validated expression difference of prognostic biomarker in transcription level between ccRCC samples and normal samples based on the Oncomine database [20] (https://www.oncomine.org/), as an external validation. In this study, four analysis were included, which were collected by the Oncomine database. We further verified the protein expression level of prognostic biomarkers between normal tissues and ccRCC tissues based on The Human Protein Atlas (HPA) database [21–23] (https://www.proteinatlas.org/).

### RNA extraction and quantitative real-time PCR (qRT-PCR)

The expression patterns of the GNRH1 and LTB4R genes were evaluated in matched ccRCC cells and normal kidney cells, ccRCC and para-cancerous tissues. The HiPure Total RNA Mini Kit and RNAiso-Plus (TAKARA, China) were used to extract total RNA from the cells and 15 pairs of kidney cancer tissue and paraneoplastic tissue which collected from the Zhongnan Hospital of Wuhan University, and we used NanoDrop to quantify the RNA which was then reverse transcribed into cDNA by ReverTra Ace qPCR RT Kit (Toyobo, China). Finally, we performed qRT-PCR analysis of cDNA with iQTM SYBR® Green Supermix (Bio-Rad) in a final volume of 20 µl. LTB4R primer: 5′-AGCTTTGTGGTGTGGAGTATCC-3′ (forward), 5′-GCAACCAGCCAGTCCAAAAC-3′ (reverse), GNRH1 primer: 5′-CAAAAACTCCTAGCTGGCCTT-3′ (forward), 5′-CAGTTGACCAACCTCTTTGACT-3′ (reverse). GAPDH primer: 5′-TGCACCACCAACTGCTTAG-3′ (forward), 5′-GATGCAGGGATGATGTTC-3′ (reverse).

### Western Blotting

The Caki1 cells and normal kidney cells were separately mixed with the lysis buffer and proteinase inhibitors to isolate total proteins. On the other hand, proteins were extracted from frozen tissues by using RIPA reagent with freshly added phosphatase and protease inhibitors (Sigma-Aldrich). SDS-PAGE gel was used to separate proteins which was then transferred to PVDF membranes (Millipore). After 2 h closed in skimmed milk, we incubated the membranes in primary and secondary antibodies respectively. The ECL western blotting detection kit (Millipore) was used to detect the resultant bands. We performed all experiments in triplicate at least. The primary antibodies included anti-GNRH1 (1:1000, Abcam), anti-LTB4R (1:1000, Abcam) and anti-GAPDH (1:1000, Abcam).

### Immunofluorescence (IF) and immunohistochemistry (IHC) staining

The cells plated in six-well pate, after washing with PBS, fixation with four per cent formaldehyde and treatment with 0.1 per cent Triton X-100, were used for immunofluorescence. After blocking with goat serum, we incubated the cells successively in the corresponding primary and secondary antibodies. Then, we visualized the nuclei of DAPI-labelled cells by confocal fluorescence microscopy. As for IHC, the paraffin sections were placed in citrate buffer for antigen retrieval and blocked in 3% H_2_O_2_. After incubated in the appropriate primary antibody and secondary antibody, the paraffin sections were blocked with the DAB chromogen solution and HRP substrate solution finally. The primary antibodies were as follows: anti-GNRH1, 1:200 (Abcam); anti-LTB4R, 1:200 (Biorbyt).

### Immune-related prognostic biomarker function analysis

In this study, for understanding the lurking functions of immune-related prognostic biomarkers, the median value of each biomarker was firstly evaluated relying on TCGA-KIRC data. In total, 530 tumor tissues were split into 2 groups (high- and low-expression groups). In the present study, “c2.cp.kegg.v6.2.symbols.gmt” was chosen as the enrichment gene set. Gene set enrichment analysis (GSEA) (http://software.broadinstitute.org/gsea/index.jsp) [24] was obtained. A signaling pathway was meaningful when nominal P < 0.05, a gene size (n) ≥ 100 and a FDR < 25% in the present part.

### Association between immune-related prognostic biomarker expression and immunocytes exploring

Immunocytes have been proved to be independent predictors of survival in cancers. The ssGSEA was a tool which could quantify the relative infiltration of 28 tumor-infiltrating immune cell types by using an enrichment score for representation of relative abundance [25]. Thus, in this study, we investigated the relationship between expression levels of selected biomarkers and immunocytes applying ssGSEA method by using R package “GSVA” [26]. Unpaired *t* test was used to explore the differences of immune cell abundance between high immune-related prognostic biomarker group and low group.

## Results

### Screening out of two prognosis-related modules

**Fig. 2:**
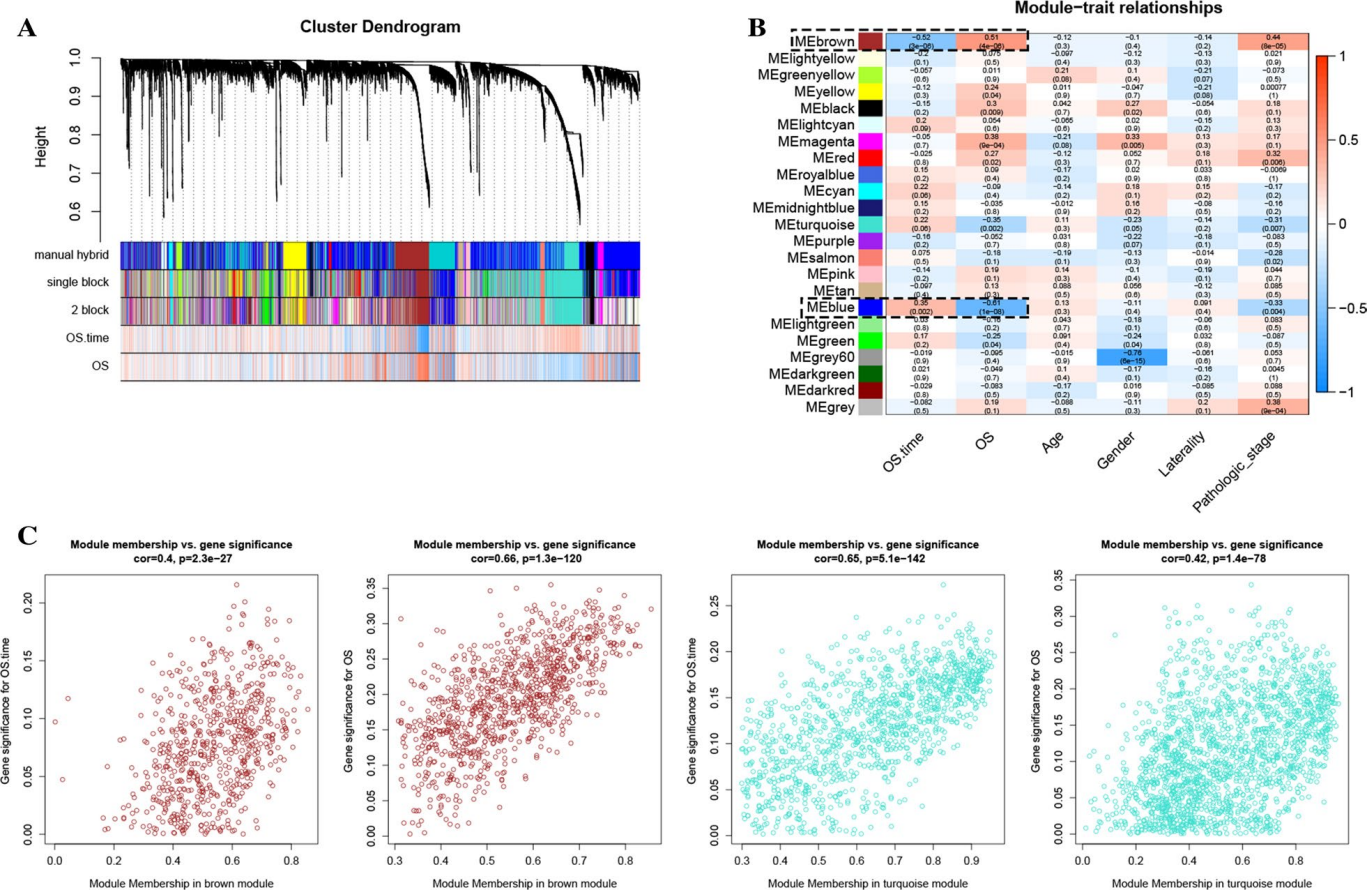
Identification of modules associated with clinical information. **A** Dendrogram of all differentially expressed genes clustered based on a dissimilarity measure (1-TOM). **B** Heatmap of the correlation between module eigengenes (MEs) and different clinical information of ccRCC (OS.time, OS, older age, gender, laterality and pathologic stage). **C** Scatter plot of module eigengenes in the brown and turquoise modules

After weeding out 21 outlier samples, 509 ccRCCs were included for WGCNA (Additional file 1: Figure S1). The beta (β) = 5 (scale free R2 = 0.84) was further set as soft-thresholding for further adjacencies calculation (Additional file 2: Figure S2). Then we split IRGs into gene modules. High-related modules were further merged for avoiding over-fitting. Eventually, as shown in Fig. 2A, totally 18 modules were identified. In this method, no-significant genes were stored in grey module, which were excluded from subsequent analysis. These genes were regarded as biomarkers with no-significance. Furthermore, brown module was associated with OS time (*P* = 0.001, R2 = − 0.14, Fig. 2B) and OS (*P* = 0.35, R2 = 0.35, Fig. 2B). Turquoise module was associated with not only OS time (*P* = 2e−05, R2 = − 0.19, Fig. 2B) but also OS (*P* = 5e−05, R2 = 0.18, Fig. 2B). The MS (of OS time) of turquoise module was higher than MSs of any other modules meanwhile the MS (of OS) of brown module was the highest compared to others (Additional file 3: Figure S3). Moreover, the associations between MM and GS for OS time (cor = 0.4, *P* = 2.3e−27)/ OS (cor = 0.66, *P* = 1.3e−120) in brown module were also significant (Fig. 2C). The same trends existed in turquoise module (OS time: cor = 0.65, *P* = 5.1e−142; OS: cor = 0.42, *P* = 1.4e−78). Both the two modules (brown, turquoise) reached the standards previous mentioned, which were considered as prognosis-related modules in the present study. We also created a network heatmap in the present study (Additional file 4: Figure S4A). As shown in Additional file 4: Figure S4B, the classical MDS plot concluded that the 18 modules were independent of each other.

### Function exploring of genes in prognosis-related modules

**Fig. 3:**
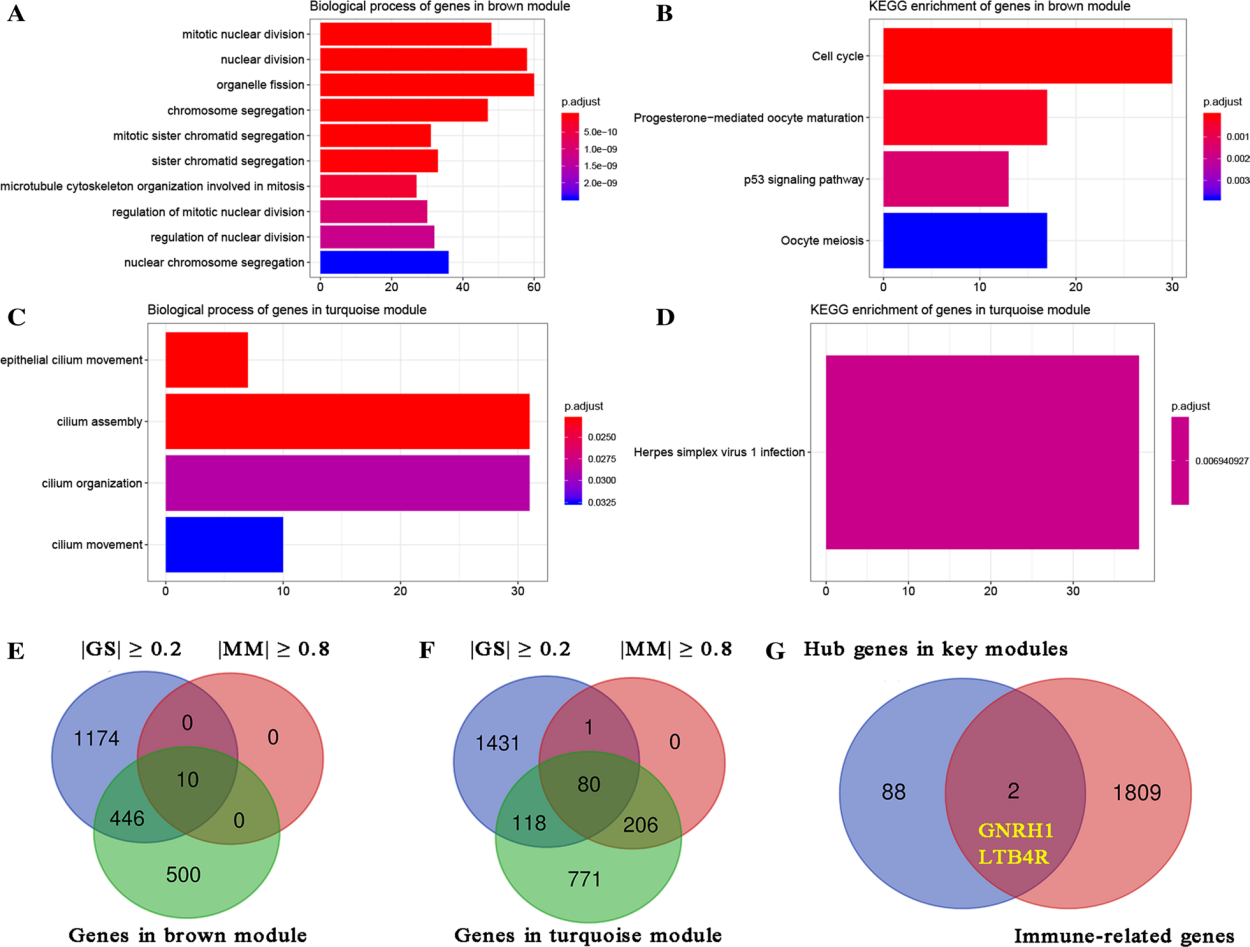
Bioinformatics analysis of genes in brown and turquoise module and identification of hub immune-related genes (IRGs) associated with prognosis of ccRCC. **A** GO analysis of genes in brown module. **B** KEGG enrichment analysis of genes in brown module. **C** GO analysis of genes in turquoise module. **D** KEGG enrichment analysis of genes in turquoise module. **E** Identification of hub genes in brown module. **F** Identification of hub genes in turquoise module. **G** Identification of hub IRGs

GO and KEGG pathway enrichment analysis were conducted to explore the potential of genes in prognosis-related modules. Genes in brown module were enriched in 106 BPs (Additional file 5: Table S1). As shown in Fig. 3A, the top 10 BPs were mitotic nuclear division, nuclear division, organelle fission, chromosome segregation, mitotic sister chromatid segregation, sister chromatid segregation, microtubule cytoskeleton organization involved in mitosis, regulation of mitotic nuclear division, regulation of nuclear division and nulcear chromosome segregation. Moreover, genes in turquoise module were mainly enriched in epithelial cilium movement, cilium assembly, cilium organization and cilium movement (Fig. 3C). The KEGG enrichment analysis indicated that genes in brown module significantly enriched in cell cycle, progesterone-mediated oocyte maturation, p53 signaling pathway and oocyte meiosis (Fig. 3B) meanwhile genes in turquoise module were mainly enriched in herpes simplex virus1 infection (Fig. 3D).

### Two genes were regarded as novel immune-related prognostic biomarkers

10 genes from brown module (Fig. 3E) and 80 genes from turquoise module (Fig. 3F) were selected by using the cut-off criterion of |cor.geneModuleMembership| > 0.8 and |cor.geneTraitSignificance| > 0.2. Finally, two genes including GNRH1 (gonadotropin releasing hormone 1) and LTB4R (leukotriene B4 receptor) overlapping in hub genes in WGCNA and IRGs were screened out (Fig. 3G).

### Internal validation of the two immune-related prognostic biomarkers

**Fig. 4:**
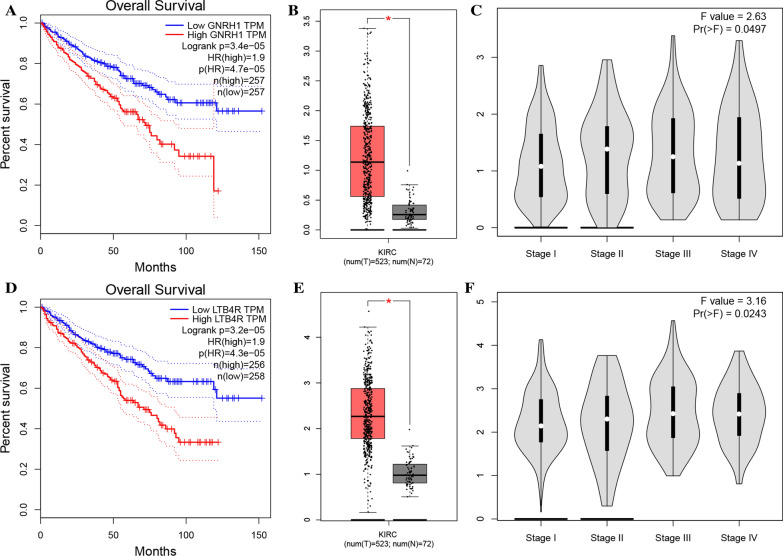
Validation of hub genes. Kaplan–Meier survival curve based on GEPIA database revealed that ccRCC patients with higher expression of hub genes had a significantly shorter overall survival time (GNRH1: **A**; LTB4R: **D**). Expressions of GNRH1 (**B**), LTB4R (**E**) in ccRCC were significantly higher than these in normal tissues based on TCGA-PAAD database (*P < 0.05). High expression of GNRH1 (**C**), LTB4R (**F**) related to higher tumor stage

After screening out four hub genes though comprehensive bioinformatics analysis, we validated these potential prognostic biomarkers. Firstly, based on the GEPIA webtool, Higher expression of GNRH1 was significantly related to worse OS (Hazard ratio [HR] = 1.9, *P* = 3.4e−05, Fig. 4A). Moreover, we concluded that patients in LTB4R high-expression group obviously occupied worse OS (HR = 1.9, *P* = 3.2e−05, Fig. 4D), accurately. Then the mRNA expression of prognostic biomarkers between tumor tissues and normal tissues was compared, the results indicated that both the genes including GNRH1 (*P* < 0.05, Fig. 4B), and LTB4R (*P* < 0.05, Fig. 4E) were significantly higher expressed in ccRCC samples compared to normal samples. Moreover, the results also suggested that high expression of GNRH1 (F = 2.63, *P =* 0.0497; Fig. 4C) and LTB4R (F = 3.16, *P =* 0.0243; Fig. 4F) were significantly related to higher tumor stage.

### Validation the prognostic value of the two immune-related prognostic biomarkers

**Fig. 5:**
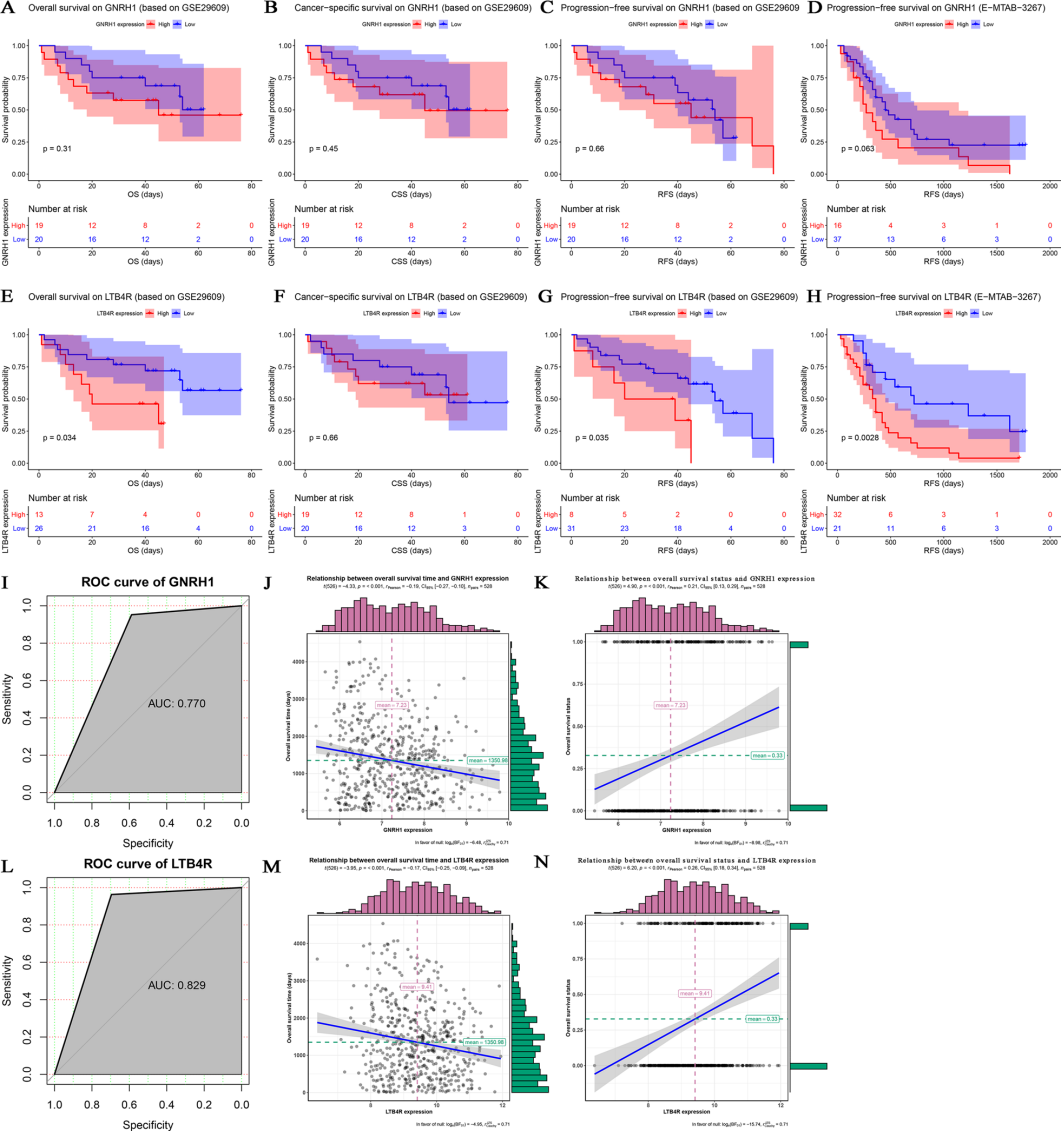
Validation of prognostic value of hub genes. Overall survival on GNRH1 by using GSE29609 (**A**). Cancer-specific survival on GNRH1 by using GSE29609 (**B**). Progression-free survival on GNRH1 by using GSE29609 (**C**) and E-MTAB-3267 (**D**). Overall survival on LTB4R by using GSE29609 (**E**). Cancer-specific survival on LTB4R by using GSE29609 (**F**). Progression-free survival on LTB4R by using GSE29609 (**G**) and E-MTAB-3267 (**H**). Prognostic value validation of GNRH1 (**I**) and LTB4R (**L**) by using ROC curve. Relationship between overall survival time and GNRH1 expression (**J**). Relationship between overall survival status and GNRH1 expression (**K**). Relationship between overall survival time and LTB4R expression (**M**). Relationship between overall survival status and LTB4R expression (**N**)

We then validated the prognostic value of these biomarkers by using other independent datasets. As the results suggested, there was a trend that higher expression of GNRH1 was significantly correlated to worse OS (GSE29609), CSS (GSE29609), PFS (GSE29609), PFS (E-MTAB-3267); suggested by Fig. 5A (*P* = 0.310), Fig. 5B (*P* = 0.450), Fig. 5C (*P* = 0.660), Fig. 5D (*P* = 0.063), respectively. Meanwhile ccRCC patients with higher LTB4R expression was related to short OS time, accurately (*P* = 0.034, GSE29609, Fig. 5E). The trend that higher expression of LTB4R was associated with worse CSS was verified by using GSE29609 (*P* = 0.660, Fig. 5F). Furthermore, ccRCC patients with higher expression of LTB4R was significantly correlated to worse PFS, as Fig. 5G (*P* = 0.035, GSE29609) and Fig. 5H (*P* = 0.0028, E-MATB-3267) suggested. In addition, we also conducted ROC analysis. The result demonstrated that GNRH1 could significantly distinguish ccRCC samples from normal samples, suggested by Fig. 5I (AUC = 0.770). Interestingly, LTB4R showed strong potential for ccRCC diagnosis (AUC = 0.829, Fig. 5L). Next-step analysis demonstrated that GNRH1 expression was significantly associated with not only OS time (*r =* − 0.19, *P* < 0.001, Fig. 5J) but also OS status (*r* = 0.21, *P* < 0.001, Fig. 5K). Moreover, LTB4R expression was significantly related to OS time (*r =* − 0.17, *P* < 0.001, Fig. 5M) and OS status (*r* = 0.26, *P* < 0.001, Fig. 5N). All the results above indicated that the two immune-related prognostic biomarkers we screened were credible.

### Validation of immune-related prognostic biomarkers and experimental validation

**Fig. 6:**
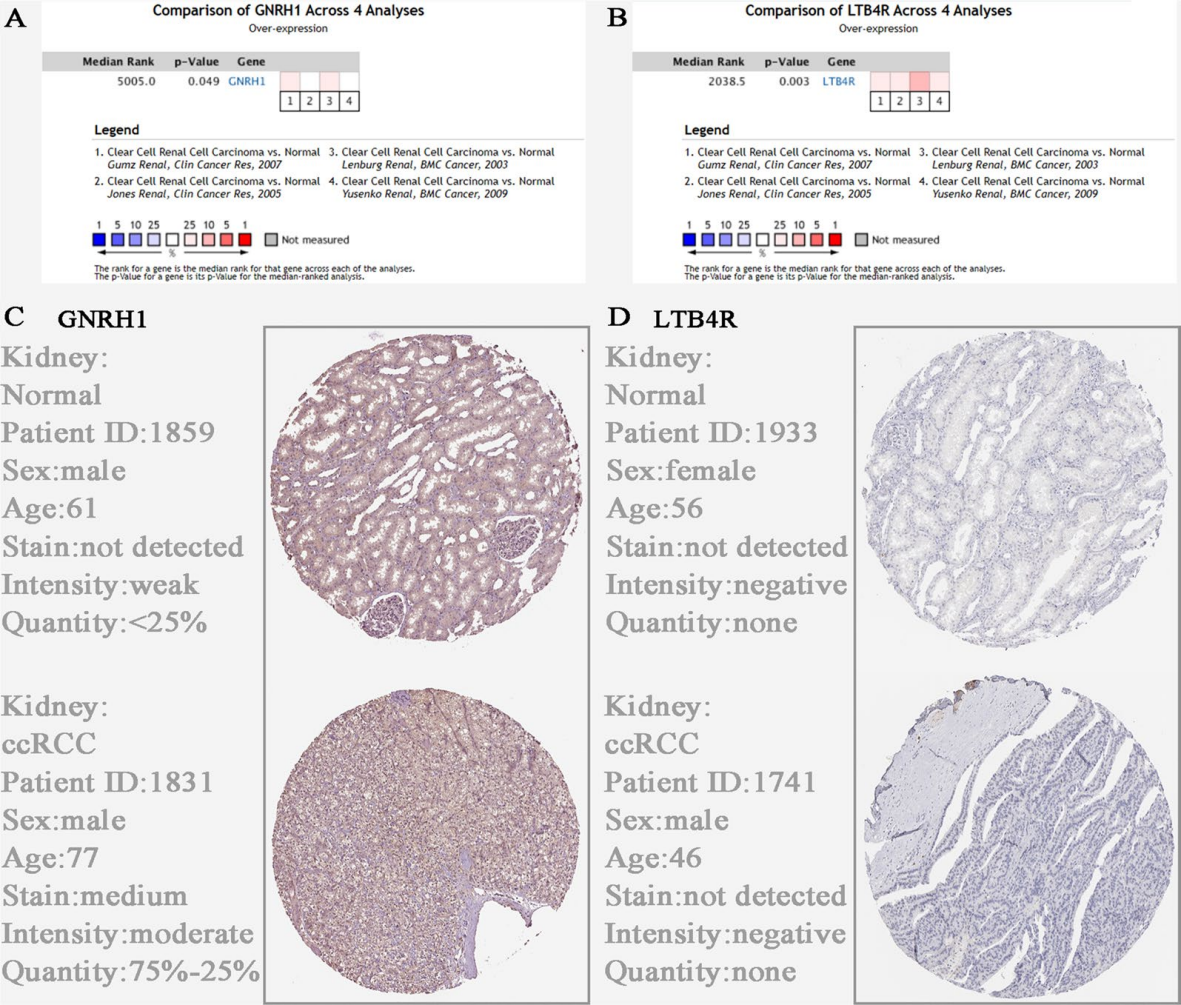
Oncomine database analyses and translational level validation of immune-related prognostic biomarkers. **A** Comparison of GNRH1 mRNA expression across 4 analyses of ccRCC based on Oncomine database. **B** Comparison of LTB4R mRNA expression across 4 analyses of ccRCC based on Oncomine database. Validation of hub gene [GNRH1 (**C**) and LTB4R (**D**)] in translational level by The Human Protein Atlas database (IHC)

**Fig. 7:**
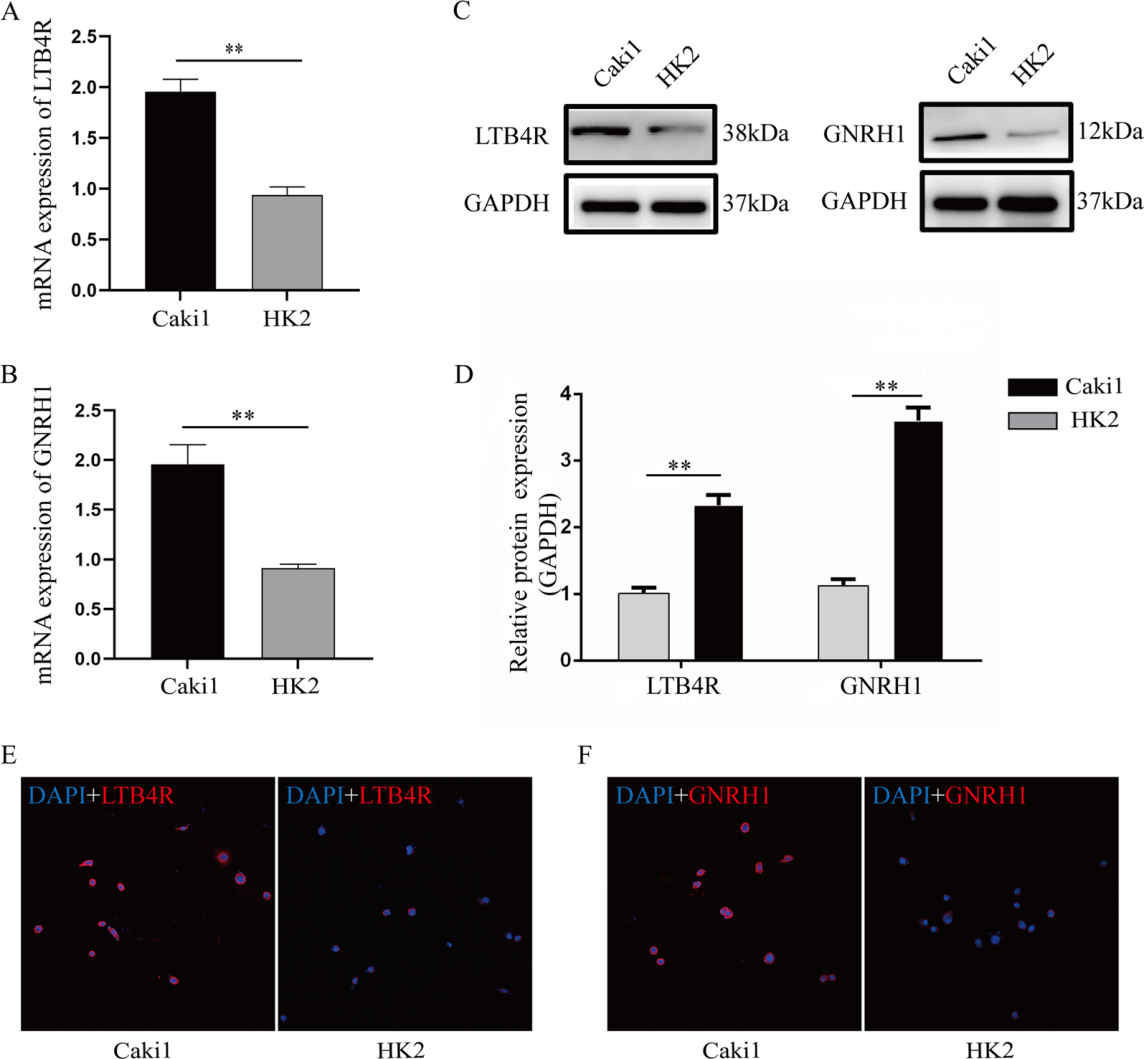
Detection of LTB4R (**A**) and GNRH1 (**B**) mRNA expression levels in ccRCC cells and normal kidney cells by qRT-PCR. GNRH1 and LTB4R expression in cancer cells is clearly higher than normal kidney cells. Overexpressed GNRH1 and LTB4R increased GNRH1 and LTB4R protein by western blot analysis (**C**, **D**). GAPDH abundance was used as an internal control. Immunofluorescence staining of LTB4R (**E**) and GNRH1 (**F**). **P < 0.01

**Fig. 8:**
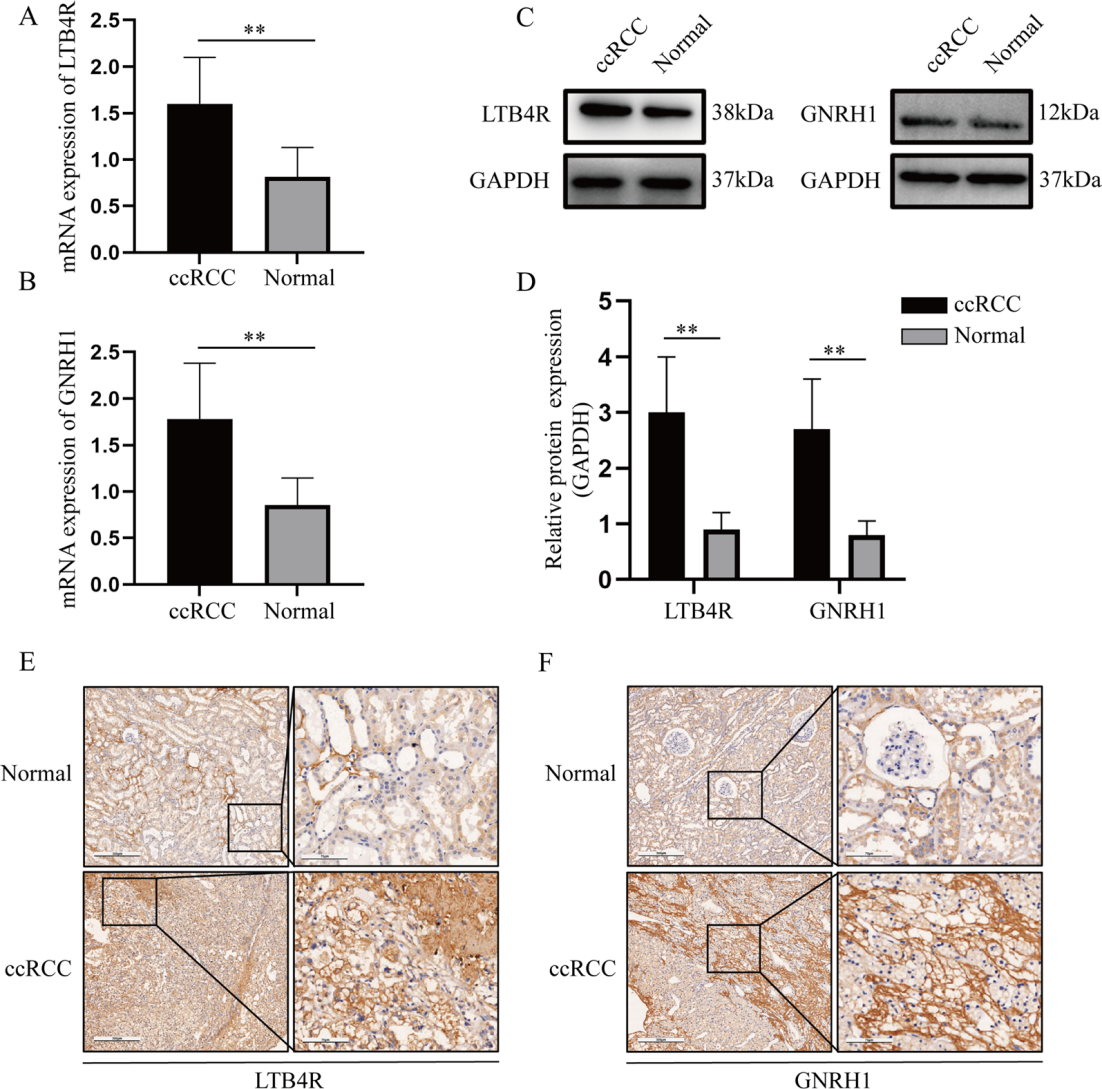
Detection of LTB4R (**A**) and GNRH1 (**B**) mRNA expression levels in 15 pairs of ccRCC and para-cancer tissues by qRT-PCR. GNRH1 and LTB4R expression in ccRCC tissues is clearly higher than para-cancer tissues. Overexpressed GNRH1 and LTB4R increased GNRH1 and LTB4R protein by western blot analysis (**C**, **D**). GAPDH abundance was used as an internal control. **E**, **F** Analyze the differential expression of LTB4R and GNRH1 in ccRCC and adjacent tissues by IHC experiments. **P < 0.01

Expression levels of prognostic biomarkers between normal samples and ccRCC samples were compared based on Oncomine database. We indicated the same conclusion that GNRH1 (*P* = 0.049, Fig. 6A) and LTB4R (*P* = 0.003, Fig. 6B) had higher expression in ccRCC tissues than in normal tissues. By using HPA database, we investigated the translational level expression of immune-related prognostic biomarkers. GNRH1 obtained medium staining in ccRCC samples compared to normal samples (not detected) (Fig. 6C). Not as we expected, there was no significant difference between ccRCC and normal tissues on the level of translation (Fig. 6D). We further validated the expression of GNRH1 and LTB4R by qRT-PCR and western blot at the cellular level and tissue level of ccRCC. qRT-PCR analysis showed that GNRH1 (*P* < 0.01, Fig. 7A) and LTB4R (*P* < 0.01, Fig. 7B) mRNA levels were significantly higher in ccRCC cells than normal kidney cells. Furthermore, compared with the normal renal epithelial cell line HK2, expression of GNRH1 and LTB4R were significantly increased in the ccRCC cell line Caki1 (Fig. 7C, D). The IF (Fig. 7E, F) also demonstrated the above conclusions. In addition, qRT-PCR analysis suggested that GNRH1 (*P* < 0.01, Fig. 8A) and LTB4R (*P* < 0.01, Fig. 8B) mRNA levels were significantly higher in ccRCC tissues than normal kidney tissues. Furthermore, compared with the normal kidney tissues, expression of GNRH1 and LTB4R were significantly increased in the ccRCC tissues (Fig. 8C, D). In addition, IHC (Fig. 8E, F) results of clinical tissue samples collected in our hospital further confirmed the above conclusions.

### Immune-related prognostic biomarker related pathways and association between immune-related prognostic biomarker expression and immune infiltration level in ccRCC

**Fig. 9:**
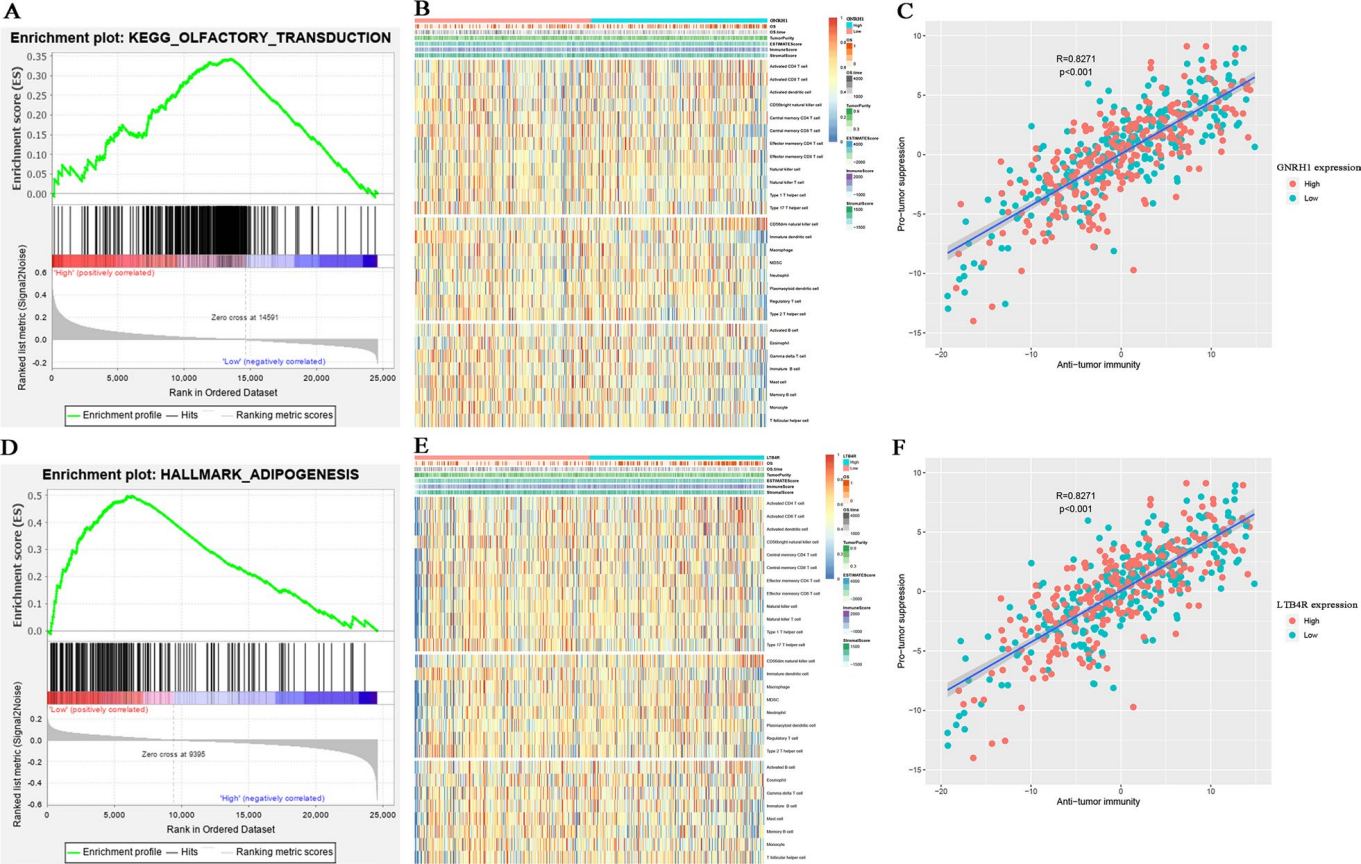
GSEA and relationship between immune-related prognostic biomarkers and immune infiltration level in ccRCC. GSEA analysis for GNRH1 (**A**) and LTB4R (**D**). **B** Heatmap for relationship of GNRH1 expression with clinical features and immune cell types. **C** Relationship between GNRH1 expression and efficacy of anti-tumor immunotherapy. **E** Heatmap for relationship of LTB4R expression with clinical features and immune cell types. **F** Relationship between LTB4R expression and efficacy of anti-tumor immunotherapy

The results of GSEA indicated that GNRH1 was significantly enriched in one KEGG signaling pathway including olfactory transduction (nominal *P* = 0.014, n = 368, FDR = 5.164%, Fig. 9A). Meanwhile, we found that LTB4R was significantly related to adipogenesis (nominal *P* = 0.035, n = 191, FDR = 22.685%, Fig. 9D). Immune infiltration was reported to be associated with survival and progression of cancers. Thus, the association between prognostic biomarker and immune infiltration level was obtained by applying ssGSEA. GNRH1 expression was significantly associated with several immune cell types, such as activated CD8 T cell (*P* < 0.01), CD56bright natural killer cell (*P* < 0.01), CD56dim natural killer cell (*P* < 0.01), central memory CD8 T cell (*P* < 0.0001), effector memeory CD8 T cell (*P* < 0.05), gamma delta T cell (*P* < 0.0001), immature dendritic cell (*P* < 0.0001), mast cell (*P* < 0.0001), MDSC (*P* < 0.05), memory B cell (*P* < 0.0001), monocyte (*P* < 0.0001), natural killer T cell (*P* < 0.05), regulatory T cell (*P* < 0.05), type 17 T helper cell (*P* < 0.01), and type 2 T helper cell (*P* < 0.01) (Fig. 9B, Additional file 6: Table S2). Moreover, LTB4R was significantly related to activated CD4 T cell (*P* < 0.05), activated CD8 T cell (*P* < 0.05), activated dendritic cell (P < 0.05), CD56bright natural killer cell (*P* < 0.05), CD56dim natural killer cell (*P* < 0.0001), central memory CD4 T cell (*P* < 0.05), immature dendritic cell (*P* < 0.0001), mast cell (*P* < 0.001), MDSC (*P* < 0.01), memory B cell (*P* < 0.0001), monocyte (*P* < 0.05), plasmacytoid dendritic cell (*P* < 0.05), type 17 T helper cell (*P* < 0.0001), and type 2 T helper cell (*P* < 0.05) (Fig. 9E, Additional file 6: Table S2). Furthermore, there was obviously strong association between anti-tumor immunity and pro-tumor suppression in ccRCC, as Fig. 9D, F displayed (*R* = 0.8271, *P* < 0.001). The results demonstrated that expression of GNRH1 and LTB4R did not influence the association. These results indicated that expression of the two prognostic biomarkers might affect the immune infiltration levels of some tumor-infiltrating immune cell types.

## Discussion

Renal cell carcinoma (RCC) is the second common malignancy after bladder cancer in the urinary system and ccRCC is the most common one representing approximately 85–90% of all renal cancer diagnosis which can occur at any age. The tumour microenvironment including various infiltrating immune cells, fibroblasts, numerous cytokines, malignant tumour cells and so on was considered a complex and dynamic ecosystem in which immune response had broad influences on tumour growth, invasion and metastasis [27]. Recent studies had shown that immune-associated genes (IAG) played an important role in the development and progression of ccRCC [28, 29]. There were some promising new treatment for ccRCC including programmed death-1 (PD-1)/programmed death-ligand 1 (PD-L1) or cytotoxic T-lymphocyte-associated protein 4 (CTLA-4) inhibitors and so on while these immunotherapies were limited by low long-term response rates and lack of reliable prognostic factors [30]. So we determined to find some novel markers related to both immunity and prognosis for diagnosis and treatment of ccRCC.

In this study, gene co-expression network was constructed on the basis of resemblance of expression profiles during samples and we explored the interconnections between genes by mean of WGCNA analysis. Meanwhile, we collected immune-related genes from various databases. By taking the intersection of the relevant results of WGCNA analysis and the data of immune genes, we got two final hub genes (GNRH1, LTB4R) which related to poor prognosis and immunity. In past studies, these two genes were reported to be correlated with prognosis of cancer. Expression of LTB4R was essential for CD8+ T cell infiltration into tumours [31] and invasiveness of ovarian cancer cells and breast cancer cells was increased by MMP-2 pathways [32] and IL-8 pathway [33] respectively. In a word, these studies highlighted the critical nature of BLT2 in tumour survival, invasiveness, chemoresistance and metastasis, as well as its potential as a therapeutic target for some cancers. During another study, expression of GNRH1 was considered may be a prognostic factor for metastatic spread of tumor cells based on the result that the up-regulation of GNRH1 expression suggested the presence of tumour cells in the circulation of cancer patients [34].

Six clinical features (OS.time, OS, Age, Gender, Laterality, Pathologic-stage) were brought into our study, and we eventually screened the 19 modules related to the prognosis of ccRCC. During them, the turquoise module had highest positively relevance to the OS.time and the brown module was most positively correlated with the OS. The turquoise module incuded 1175 genes and the brown module included 956 genes. We ended up with a total of 90 hug genes from these two modules. On the other hand, we got the immune-related genes from ImmPort database which included 1811 genes. We found out the real hub genes which common to the turquoise module, the brown module and the Immune related genes. Two final hub genes (GNRH1, LTB4R) related to poor prognosis of ccRCC were ensured and we also investigated the correlation between final hub genes, clinical features and immune cells. In order to further investigate the mechanism of these genes in regulating tumor genesis, GSEA analysis was performed by using TCGA-KIRC data from TCGA database. Finally, we used various methods to validate the hub genes internally and externally respectively. However, the biggest limitation for our study was that we did not validate these two genes in vivo and in vitro. We need to verify this at the cellular, tissue and animal level and so on. Moreover, although this study predicted that the immune-related prognostic biomarkers involved in cell cycle regulation, more-depth studies were need to explore the immunological function of hub genes and regulation mechanism of these genes to immune responses.

## Conclusions

In summary, this study used a variety of bioinformatics analysis methods to identify two new hub genes that might play an important role on the occurrence, development and prognosis of ccRCC. At the same time, we predicted the potential function of hub genes involved in cell cycle regulation, which are associated with staging and prognosis. The two hub genes may become novel biomarkers of ccRCC in human. However, this study only selected potential ccRCC biomarkers related to tumor development and prognosis and the effect of candidate biomarkers need to be verified by further molecular biology experiments.

## Supplementary Information

**Additional file 1: Figure S1.** **A** Sample clustering to detect outliers. **B** Sample dendrogram and trait heatmap. The color intensity was proportional to OS.time, OS, Age, Gender, Laterality, and Pathologic stage.

**Additional file 2: Figure S2.** Determination of soft-thresholding power in the weighted gene co-expression network analysis (WGCNA). **A** Analysis of the scale-free fit index for various soft-thresholding powers (β). **B** Analysis of the mean connectivity for various soft-thresholding powers. **C** Histogram of connectivity distribution when β = 5. **D** Checking the scale free topology when β = 5.

**Additional file 3: Figure S3.** Diagram of correlation of module’s color and ccRCC. The relevance between eigenvalue of network modules and prognosis of ccRCC was qualified. The colored row indicates modules and the Y-axis represents gene significance. **A** Represents the module significance of OS.time. **B** Represents the module significance of OS.

**Additional file 4: Figure S4.** Interaction relationship analysis of co-expression genes and construction of a classical MDS plot. **A** Different colors of horizontal axis and vertical axis represent different modules. The brightness of yellow in the middle represents the degree of connectivity of different modules. There was no significant difference in interactions among different modules, indicating a high-scale independence degree among these modules. **B** Classical MDS plot whose input is the TOM dissimilarity. Each dot (gene) is colored by the module assignment.

**Additional file 5: Table S1.** GO biological processes of genes in brown module.

**Additional file 6: Table S2.** Significance of relationship of GNRH1/LTB4R expression and immune cell types. ns no significance, * *P* < 0.05, ** *P* < 0.01, *** *P* < 0.001, **** *P* < 0.0001.

## Data Availability

The data used to support the findings of this study are available from the corresponding author upon request.

## Abbreviations

ccRCC: Clear cell renal cell carcinoma
GSEA: Gene set enrichment analysis
ROC: Receiver operating characteristic curve
HPA: The Human Protein Atlas
ANOVA: Analysis of variance
TCGA: The Cancer Genome Atlas
GEO: Gene Expression Omnibus
WGCNA: Weighted gene co-expression network analysis
IRG: Immune-related genes
GS: Gene significance
MS: Module significance
MEs: Module eigengenes
OS: Overall survival status
OS time: Overall survival time
GO: Gene Ontology enrichment analysis
KEGG: Kyoto Encyclopedia of Genes and Genomes enrichment analysis
DFS: Disease-free survival
PAAD: Pancreatic adenocarcinoma
GEPIA: Gene expression profiling interactive analysis
GSVA: Gene set variation analysis
HR: Hazard ratio
