# Supplementary figures and images for: GNRH1 and LTB4R might be novel immune-related prognostic biomarkers in clear cell renal cell carcinoma (ccRCC)
Source: Cancer Cell Int. 2021 Jul 6;21:354. doi: 10.1186/s12935-021-02052-1 (PMC8259034; doi:10.1186/s12935-021-02052-1)

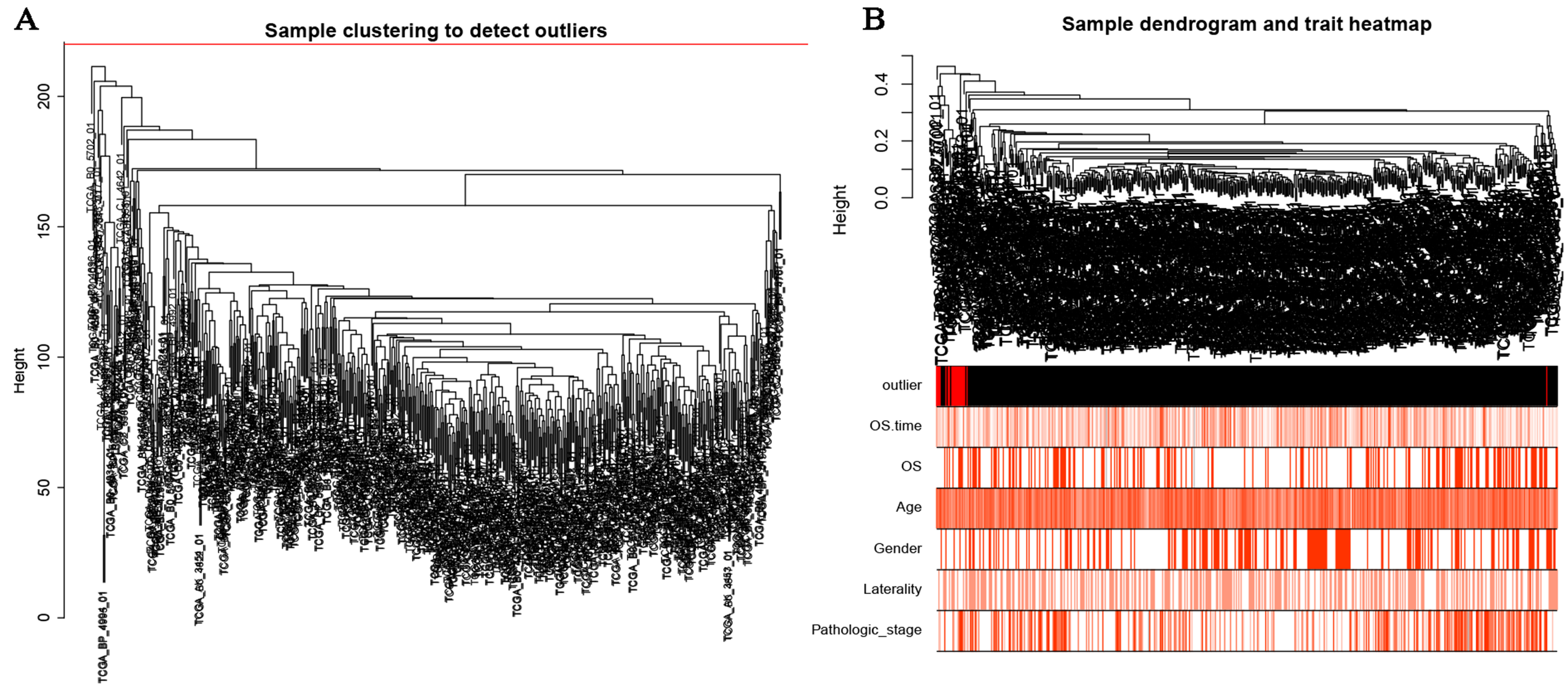

Supplement: Supplementary file 1 — Additional file 1: Figure S1. A Sample clustering to detect outliers. B Sample dendrogram and trait heatmap. The color intensity was proportional to OS.time, OS, Age, Gender, Laterality, and Pathologic stage. [file 12935_2021_2052_MOESM1_ESM.tif]

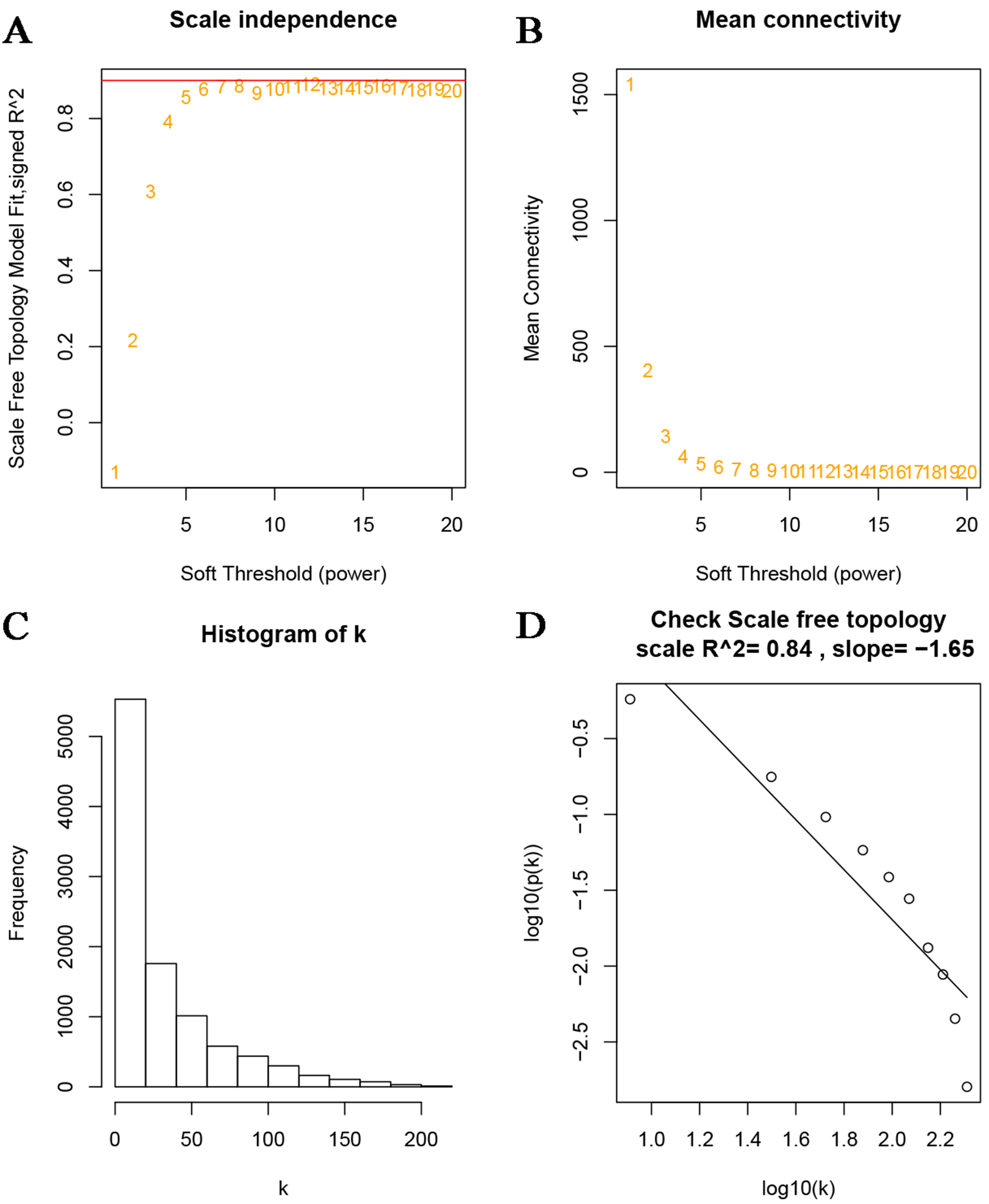

Supplement: Supplementary file 2 — Additional file 2: Figure S2. Determination of soft-thresholding power in the weighted gene co-expression network analysis (WGCNA). A Analysis of the scale-free fit index for various soft-thresholding powers (β). B Analysis of the mean connectivity for various soft-thresholding powers. C Histogram of connectivity distribution when β = 5. D Checking the scale free topology when β = 5. [file 12935_2021_2052_MOESM2_ESM.tif]

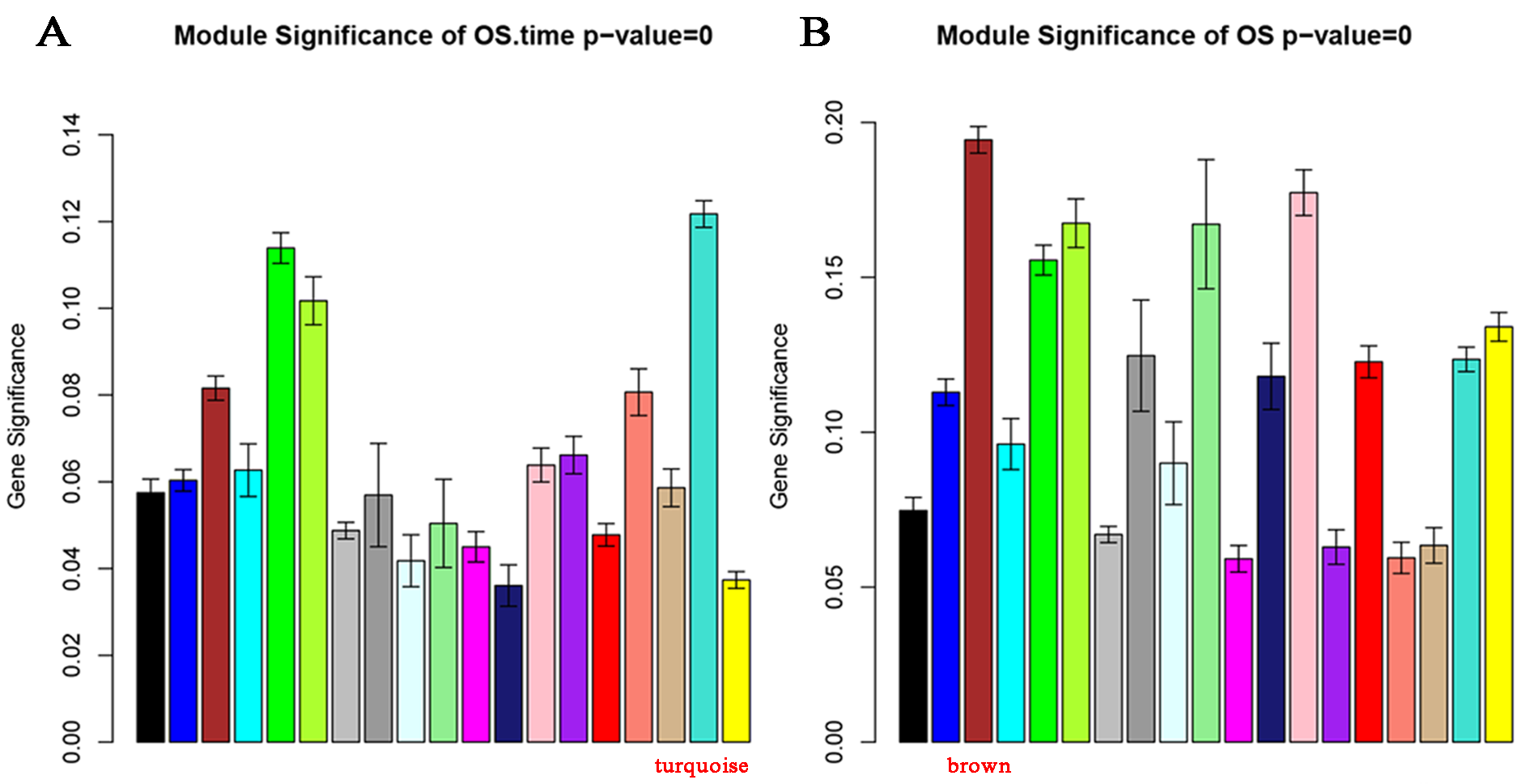

Supplement: Supplementary file 3 — Additional file 3: Figure S3. Diagram of correlation of module’s color and ccRCC. The relevance between eigenvalue of network modules and prognosis of ccRCC was qualified. The colored row indicates modules and the Y-axis represents gene significance. A Represents the module significance of OS.time. B Represents the module significance of OS. [file 12935_2021_2052_MOESM3_ESM.tif]

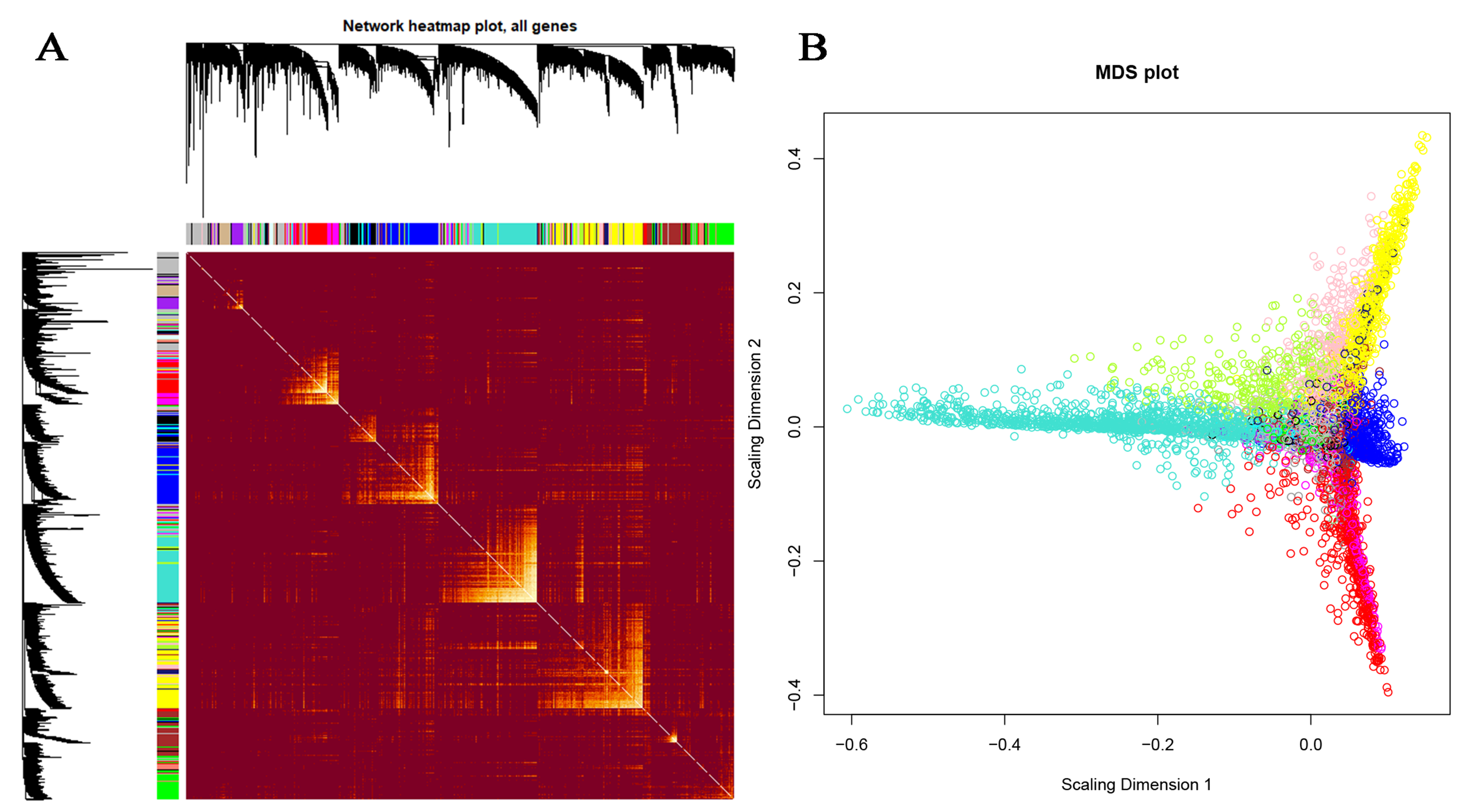

Supplement: Supplementary file 4 — Additional file 4: Figure S4. Interaction relationship analysis of co-expression genes and construction of a classical MDS plot. A Different colors of horizontal axis and vertical axis represent different modules. The brightness of yellow in the middle represents the degree of connectivity of different modules. There was no significant difference in interactions among different modules, indicating a high-scale independence degree among these modules. B Classical MDS plot whose input is the TOM dissimilarity. Each dot (gene) is colored by the module assignment. [file 12935_2021_2052_MOESM4_ESM.tif]
